# Handgrip Strength and Upper Limb Anthropometric Characteristics among Latin American Female Volleyball Players

**DOI:** 10.3390/jfmk9030168

**Published:** 2024-09-18

**Authors:** María Alejandra Camacho-Villa, Jhon Hurtado-Alcoser, Andrés Santiago Jerez, Juan Carlos Saavedra, Erika Tatiana Paredes Prada, Jeimy Andrea Merchán, Fernando Millan-Domingo, Carlos Silva-Polanía, Adrián De la Rosa

**Affiliations:** 1 Laboratory of Exercise Physiology, Sports Science and Innovation Research Group (GICED), Unidades Tecnológicas de Santander (UTS), Bucaramanga 680006, Colombia; mcamacho@correo.uts.edu.co (M.A.C.-V.); jsaavedra@correo.uts.edu.co (J.C.S.); eparedes@correo.uts.edu.co (E.T.P.P.); jandreamerchan@correo.uts.edu.co (J.A.M.); fernando.millan-domingo@uv.es (F.M.-D.); 2 Pain Study Group (GED), Physical Therapy School, Universidad Industrial de Santander, Bucaramanga 680002, Colombia; 3 Physical Activity and Sport Program, Sports Science and Innovation Research Group (GICED), Unidades Tecnológicas de Santander (UTS), Bucaramanga 680006, Colombia; jandersonhurtado@uts.edu.co (J.H.-A.); asjerez@uts.edu.co (A.S.J.); 4 Freshage Research Group, Department of Physiology, Faculty of Medicine, University of Valencia, CIBERFES, Fundación Investigación Hospital Clínico Universitario/INCLIVA, 46010 Valencia, Spain; 5 Body, Physical Activity and Sport Study Group (GECAFD), Sports Department, Universidad Industrial de Santander, Bucaramanga 680002, Colombia; carlos2248261@correo.uis.edu.co

**Keywords:** grip strength, female athletes, hand dimensions, talent selection

## Abstract

**Background:** In volleyball, the upper limb dimensions and grip strength greatly influence offensive and defensive movements during a match. However, the relationship between these parameters remains underexplored in elite female volleyball players. **Objective:** This study aimed to contrast the upper limb anthropometric characteristics and handgrip strength (HGS) of female elite volleyball players against a control group. **Methods:** Selected upper limb anthropometric parameters and maximal HGS of 42 female volleyball players and 40 non-athletes were measured. **Results:** Players exhibited higher values in almost all variables studied than non-athletes. The differences were statistically significant (*p* < 0.001) except for body mass index and elbow and wrist diameters. Players showed a moderate correlation between dominant HGS and hand parameters (length r = 0.43 and breadth r = 0.63; *p* < 0.05). Weak correlations were identified with height, upper arm length, elbow diameter, and hand shape index (r = 0.32 to 0.38; *p* < 0.05). In the non-dominant hand, a moderate correlation with handbreadth (r = 0.55, *p* ≤ 0.01) and weak correlations with upper arm length, wrist diameter, hand length, and hand shape index (r = 0.32 to 0.35; *p* ≤ 0.05) was found. **Conclusions:** These findings underscore the importance of the upper limb anthropometric parameters as predictors of HGS and their utility in athlete selection. Future research should investigate biomechanical factors influencing HGS and injury prevention.

## 1. Introduction

Volleyball sport requires several high-intensity and high-velocity actions combined with explosive exertions interspersed with short resting intervals [1]. During the game, success largely depends on motor abilities, particularly muscle strength conditioning, both in the lower and upper limbs. In elite female matches, technical actions that produce the highest score rely on the continuous engagement of the wrist and digit flexor, including attack (76.8–80%), block (14.5–15.6%), and serve (4.4–8.1%) [2,3]. Consequently, upper extremity and grip strength are fundamental to the sport, being the primary physical factors influencing these specific movements.

Additionally, some anthropometric measurements and morphological characteristics (e.g., height, weight, body composition, arm, and hand dimensions) impact the player’s performance across this game, making all shots and passes work more efficiently when there is a larger hand surface and longer, stronger fingers [4,5]. For this reason, handgrip strength (HGS) and anthropometric dimensions have been investigated in other popular sports, such as basketball, softball, and handball, where the relationship between hand and ball is fundamental [6]. Nevertheless, in female volleyball players, research has extensively reported on the relationship between the strength of the lower limbs and volleyball success [7,8], with few authors investigating anthropometric and muscle strength in the upper limbs. For instance, Khanna and Koley found higher values in HGS, height, hand, and arm anthropometrics compared to the reference group (*p* < 0.05) [4]. Additionally, Koley and Kaur reported weak to moderate positive correlations (r = 0.28 to 0.48) between upper limb anthropometric variables (i.e., arm length, hand breadth, and hand length) and HGS among Indian inter-university female volleyball players [9].

HGS test has been extensively used to assess upper limb strength across various sports, with high levels of HGS identified as a critical factor for success. Recently, the relationship between HGS and serve reception efficiency has been reported in volleyball players, indicating that HGS is a key element in achieving success during games. Similarly, moderate correlations have also been reported between HGS and both the velocity of serving and spike [10,11]. These findings indicate that HGS assessment is a valuable tool for identifying talent, strengths, and weaknesses in the physical condition of volleyball players. Moreover, it is considered a non-invasive and cost-effective method for collecting extensive data [12].

Several anthropometric characteristics of the upper limbs are different across sports in female athletes. Thus, while hand breath and hand length were greater in basketball collegiate athletes as compared to the handball ones [6], these measurements, along with the forearm length and forearm circumference, were reported to be greater in a group of athletes as compared to non-athletes (national basketball players, collegian handball players, collegian volleyball players, and collegian wrestlers) [13]. Similarly, when comparing anthropometric measurements of elite volleyball players and non-athletes, researchers found that an athlete’s hand measurements, such as hand length and hand finger length, but not hand width, were greater in the dominant hand [14]. Therefore, hand dimensions, including the aforementioned, are interrelated and have been described to significantly contribute to the techniques applied in grappling sports such as volleyball [13].

Although several studies have emphasized the link between upper limb anthropometric variables, HGS, and the specific skills required for volleyball players [9,11,14], the interplay between hand, forearm, and arm-anthropometric variables with HGS in elite female volleyball players remains largely unreported. Moreover, the influence of the practice of volleyball in HGS and some upper limbs anthropometric variables is unknown.

Hence, this study has two aims: (i) To contrast the upper limb anthropometric characteristics and HGS of female Latin American elite volleyball players against a control group, and (ii) To determine the relationship between these variables in female volleyball players. The present study has two hypotheses: (i) Latin American female volleyball players will have greater upper limb dimensions and HGS than the controls, and (ii) HGS will correlate with upper limb anthropometric variables.

## 2. Materials and Methods

### 2.1. Participants

A cross-sectional analytical study was conducted during the “International Cup Ciudad de Bucaramanga”, which took place in July 2022 in Bucaramanga-Colombia. Forty-two female volleyball players belonging to the national teams of Chile (*n* = 13), Colombia (*n* = 17), and Mexico (*n* = 13) were examined (age: 24.63  ±  5.31 yrs; height: 1.68  ±  0.04 m; weight: 67.26  ±  8.46 kg; years of volleyball experience: 10.45 ± 5.14 yrs). The control group was comprised of forty non-athlete young females (age 22.81  ±  2.27; body height 158  ±  0.06 cm; body weight 57.61  ±  5.99 kg). These participants were physically inactive university students from Unidades Tecnológicas de Santander in Bucaramanga, Colombia.

All the participants were informed of the purposes and content of the study; written informed consents were obtained from each player and woman in the control group. The research complied with the Helsinki Declaration and the protocol was approved by the Ethics Committee for Human Beings from the Unidades Tecnológicas de Santander, no. 0010-2022/02.05.2022.

### 2.2. Selection Criteria

The inclusion criteria were as follows: (a) for the control group, do not report more than 150 weekly minutes of moderate-intensity physical exercise (<600 METS-min/week) in the short version of the International Physical Activity Questionnaire (IPAQ); (b) free of any neuromuscular, orthopedic, or neurological conditions that might interfere with their sports performance, hand function, anthropometric characteristics and activities of daily living.

### 2.3. Procedures

For volleyball players, all data were collected before training in a private room in the Bicentenario Volleyball Coliseum under natural environmental conditions in the morning (between 8:00–11:00 a.m.).

Regarding the control group, data were collected throughout the same month at the sports science laboratory of the Unidades Tecnológicas de Santander under the same conditions as volleyball players. The entire sampling was assessed by the same two researchers with nine years of experience in sports research. Evaluations were conducted in the following order: body composition, upper limb anthropometric variables and finally the Handgrip strength assessment. Finally, to standardize the measurement technique of HGS, the investigator underwent training which included the participant’s position and verbal encouragement.

### 2.4. Body Composition and Anthropometric Parameters

All the assessments were conducted by a level 2 anthropometrist, following the international standards for anthropometric assessment published by the International Society for the Advancement of Kinanthropometry—ISAK. For data analysis, the averages of two measurements of each anthropometric variable were calculated and processed.

Height was measured with the participants in bare feet using a mechanical stadiometer platform (Seca^®^ 274, Hamburg, Germany; TEM = 0.019%). The movable headpiece was brought down to touch the top of their heads during deep inhalation, and the measurements were recorded in centimeters and rounded to the nearest 0.5 cm.

For body composition evaluation, a bioelectrical impedance device was used (TANITA BC 240, Tokyo, Japan), with measurements rounded to the nearest 0.1. Before the measurement, athletes were required not to carry metal objects, not to consume any caffeine or diuretics in the previous 3 h, and to urinate within 30 min before the test. The data collected included body mass (BM), body fat percentage (BF%), and total body water (BW%). Body mass index (BMI) was calculated as the ratio between weight and the square of height (kg/m^2^), representing the easiest method to calculate any state of underweight (<18.5 kg/m^2^), normal weight (18.5 to 24.9 kg/m^2^), overweight (25 to 29.9 kg/m^2^), or obesity (≥30 kg/m^2^) [15].

### 2.5. Measurements of Upper Limbs Anthropometric Parameters

**Figure 1:**
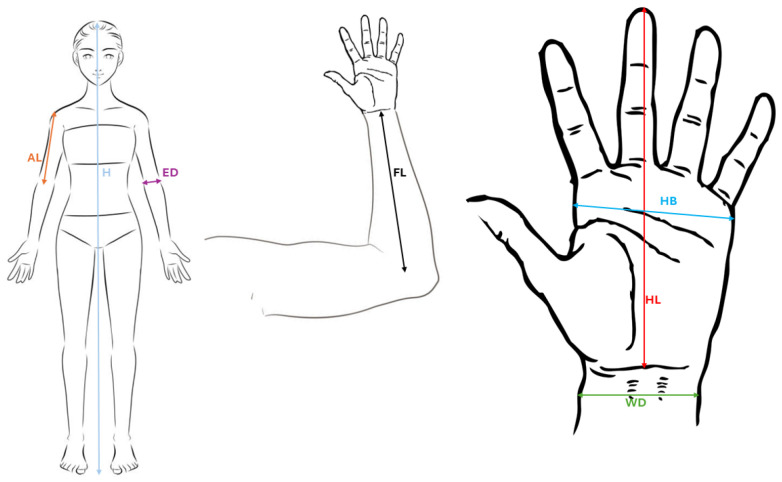
(AL) Arm Length. (H) Height. (ED) Elbow Length. (FL) Forearm Length. (WD) Wrist Diameter. (HL) Hand Length. (HB) Hand Breadth.

All the anthropometric measurements were taken, with the participants wearing minimal clothing and no shoes. For each upper limb, arm and forearm length, along with three parameters related to hand dimensions, were evaluated (handbreadth, hand length, and hand shape index) [4] as shown in Figure 1. A segmometer and a small bone anthropometer (Cescorf, Porto Alegre, Brazil) were used to measure lengths and diameters, respectively. All the upper limb measurements were taken to the nearest 0.1 cm.

The anatomical references of selected anthropometric upper limb parameters were as follows:

- **Arm length:** the distance from the marked acromial to the marked radiale. The subject stands erect with the arms at the sides and palms against the thighs.
- **Forearm length:** the distance from the marked radiale to the marked stylion. The elbow is flexed, and the orientation of the tape is such that it parallels the long axis of the radius.
- **Elbow diameter:** this is the distance between the medial and lateral epicondyles of the humerus.
- **Wrist diameter:** the distance between the outer borders of the radial and ulnar styloid processes.
- **Hand length:** the measurement is taken as the shortest distance from the marked mid-stylion line to the Dactylion.
- **Handbreadth:** the distance between the radial side of the second metacarpal joint to the ulnar side of the fifth metacarpal joint.
- **Hand Shape index:** the handbreadth and length ratio multiplied by a hundred.

### 2.6. Measurements of Handgrip Strength

The maximal HGS was measured in both hands with a portable digital hand dynamometer (Takei 5401; Tokyo, Japan) with a precision of 0.1 kg. During the hand strength testing protocol, the participants maintained an upright posture with the shoulder of the test arm adducted and the elbow flexed at 90°. The forearm and wrist were kept in a neutral position, and the hand was aligned with the forearm holding the instrument. The dynamometer was adapted to each subject, fitting the hand and allowing flexion at the metacarpophalangeal joints. Specific verbal instructions were provided to the subjects before the evaluations, and verbal encouragement was given during the experiments [16,17].

The participants performed three maximum voluntary contractions for 5 s on each side, with a 60 s rest break between each trial. The subjects were instructed to squeeze the dynamometer as hard as possible. The scale of the dynamometer indicated HGS in kilograms (kg). For statistical analyses, the highest strength value from the three tests of each hand was used [18,19].

### 2.7. Statistical Analysis

All data were examined for normality of distribution using the Shapiro–Wilk and Ladder of Powers test. Descriptive, parametric, and non-parametric statistical analyses were performed with Stata 13 (StataCorp 2013). Depending on their distribution, sample descriptive values are presented as mean ± standard deviation or median ± interquartile range (IQR).

Comparisons between two independent samples (controls vs. volleyball players) in all variables were performed with the Student’s *t*-test or Mann-Whitney test [20]. Cohen’s d value was used to evaluate effect size (ES) for the independent nonparametric and parametric analyses. The effect size was interpreted using the following conventions: small effect (d ≥ 0.20), medium effect (d ≥ 0.50), and large effect (d ≥ 0.80) [21].

Pearson’s and Spearman’s correlation coefficients were used to establish the magnitude of the correlations between dominant and non-dominant HGS and anthropometric variables in volleyball players [22]. According to Schober et al. (2018) [22], the conventional approach to interpreting a correlation coefficient is to categorize it as “negligible” (r = 0.00–0.10), “weak” (r = 0.10–0.39), “moderate” (r = 0.40–0.69), “strong” (r = 0.70–0.89) and “very strong” (0.90–1.00).

In all analyses, a *p*-value of less than 0.05 was considered a statistically significant result.

## 3. Results

The right hand was identified as the dominant hand in 81.70% (*n* = 67) of the 82 participants. Table 1 shows the descriptive statistics of the sample, including anthropometric characteristics and HGS in both upper limbs. Latin-American female volleyball players exhibited higher values in almost all variables studied, except for body fat, than their control counterparts. These differences were statistically significant (*p* < 0.001) except for BMI and elbow and wrist diameter in both upper limbs.

Table 2 presents the correlations between anthropometric variables and dominant HGS in controls and Latin-American volleyball players. The control group showed a moderate positive correlation with forearm length and elbow and wrist diameter (r = 0.44 to 0.47; *p* ≤ 0.05). Additionally, weak positive correlations were demonstrated for upper arm length, hand length, and hand breadth (r = 0.34 to 0.39; *p* ≤ 0.05). Interestingly, only the female volleyball players showed a moderate positive correlation between dominant HGS and hand parameters (length r = 0.43 and breadth r = 0.63; *p* ≤ 0.05). Furthermore, weak positive correlations were identified with height, upper arm length, elbow diameter, and hand shape index (r = 0.32 to 0.38; *p* ≤ 0.05).

In addition, Table 3 presents the correlations between anthropometric variables and non-dominant HGS in controls and volleyball players. In the control group, only weak correlations were observed with upper arm length, elbow diameter, and hand breadth (r = 0.35 to 0.38; *p* ≤ 0.05). In contrast, volleyball players exhibited a moderate positive correlation with handbreadth (r = 0.55, *p* ≤ 0.01) and weak positive correlations with upper arm length, wrist diameter, hand length, and hand shape index (r = 0.32 to 0.35; *p* ≤ 0.05).

## 4. Discussion

In this study, we have provided an overview of upper limb anthropometric characteristics and HGS of female volleyball players belonging to different National Teams in Latin America. Among the main findings, significant differences (*p* < 0.05) were found in all the lengths and breadths of both dominant and non-dominant upper limbs between volleyball players and non-athletes, with athletes exhibiting the largest measurements. In addition, we found an association between most of the hand, forearm, and arm dimensions with HGS.

Volleyball players are required to constantly develop their muscle strength, technique, and tactics to improve their performance during a match. Most researchers have mainly focused on the analysis of lower limb strength and body composition of male athletes. In this sense, the study of anthropometric and muscle strength in the upper limbs of female volleyball players has received less attention.

Several studies have described the importance of anthropometric profiles and HGS across sports [23,24,25,26,27,28]. In the present study, elite volleyball players were taller, had a lower body fat percentage, and exhibited higher values in most of the selected upper limb variables than the control group (Table 1). In addition, statistical differences were also found in HGS performance on both sides, with players being stronger than controls (Table 1). Greater height, larger body dimensions, and enhanced HGS have been previously identified as key factors contributing to success in volleyball [29,30,31,32,33].

Recent research on female university students in Poland, with at least five years of volleyball training experience, revealed similar results in wrist diameter, height, and BMI. However, they reported lower values in some upper limb anthropometric variables compared to our study, suggesting that this may be a differentiating factor related to the level of expertise in this sport [34].

In a study conducted by Fallahi and Jadidian [13], the authors compared different anthropometric upper limb dimensions and HGS in a group of athletes (national basketball players, collegian handball players, collegian volleyball players, and collegian wrestlers) and non-athletes. The researchers found greater HGS values in players, which were accompanied by higher measurements in hand length, palm length, palm width, forearm length, forearm circumference, and wrist circumference. In addition, most of the upper limb dimensions were positively correlated with HGS [13]. Despite the analysis not considering each sport separately, these results show that athletes who have handgrip movements with an object or opponent have specific characteristics that deserve to be trained or considered in talent identification.

Likewise, in female volleyball players of the Turkish league, Öcal et al. [14] reported higher hand dimensions when those were compared with non-athletes. Interestingly, researchers found statistical differences in only two of three selected hand measurements (hand length and hand finger length) and no differences in height. Despite the level of the athletes described in this research, these partial differences could have been due to a non-strict control group selection, as the authors did not report it.

Similar results to those reported in our study have been found by other researchers [9,29]. These studies compared upper limb dimensions and HGS between inter-university female volleyball players and a control group with no particular athletic background. Statistical differences were evidenced in the left-hand width and the lengths of the hand, forearm, and arm on both sides, with volleyball players exhibiting higher values. Moreover, volleyball players displayed higher HGS scores on both hands. Despite differences in the athletic level of the population as compared to those evaluated in our study, the results were similar and suggest an overall tendency for greater upper limb dimensions in volleyball players.

In our study, athletes were also stronger and taller, and they showed the largest measurements in hand length, hand breadth, and arm length as compared to those reported in Koyle’s studies [9,29]. These differences could be explained, in part, by the fact that athletes in our study were the result of an exhaustive selection by national team coaches.

The latest findings from Sarafyniuk et al. [35] in 108 female volleyball players aged 16 to 20 found larger girth sizes in the upper limbs of athletes compared to the control group. Nevertheless, the authors did not report the years of training and level of expertise of the volleyball players, which may be crucial variables to explain the plausible differences between groups.

HGS scores in our study were also higher than those reported in other studies on volleyball players [4,6] and different sports in female athletes, ranging from 24.7 to 26.5 kg. These differences could be attributed to the specific characteristics of the participants in our study, who are elite volleyball players. Building on that point, it is important to note that a greater amount of strength in both hands is crucial for many offensive and defensive actions during a match, such as serving, passing, spiking, and blocking. For instance, while Pawlik et al. [11] reported moderated and large correlations between HGS in both hands and serve reception efficiency, and Novianingsih and Irianto [36] studied the influence of the hand’s muscle strength on the volleyball float serve skill, in adolescent volleyball players. After the assessment, researchers reported more accuracy (*p* < 0.05) in the float serve in those who had higher hand strength.

With respect to correlations, in our study, anthropometric variables such as height, upper arm length, elbow diameter, and hand dimensions (length, breadth, and shape index) showed weak to moderate correlations with dominant HGS values in volleyball players. Similarly, in non-dominant HGS, upper arm length, wrist diameter, and hand dimension showed the same range of magnitude of correlation. Our findings underline that although height is considered one of the most important physical characteristics in volleyball players, this anthropometric measurement showed only a weak positive correlation with dominant HGS (Table 2). Similar findings have been reported by Pizzigally L. et al. [16] in Italian female basketball players, where the correlation reported between height and HGS was also weak (r = 0.38, *p* < 0.05).

In addition to height, arm length, hand length, hand breadth, and hand shape index should also be considered important parameters in the process of talent identification for female volleyball players. These anthropometric characteristics are crucial for increasing HGS and enhancing sports performance, given the numerous specific movements in volleyball, where the hand is the only point of physical contact between the athlete and the ball [16,32,37].

Regarding hand dimensions, in ball sports such as volleyball, players with larger hands and longer fingers have greater performance and accuracy in the blocking, spiking, and service techniques during the match [13]. Considering the positive association between hand measurements and HGS in both upper limbs found in our study, athletes with these features may possess the ability to apply greater force to the ball and perform a combination of finely controlled movements in defensive and offensive maneuvers that contribute to competitive success [13,32].

Additionally, the timing and sequencing of the force applied to an object (i.e., ball) by the hand depends on several factors including technique, strength, flexibility, and anthropometry [32]. Our results confirm that athletes with specific body anthropometric values such as height, arm length, and hand dimension, may have biomechanical advantages to achieve higher values of HGS.

Grip strength is produced by the joint contraction of the flexor and extensor muscles of the wrist (maintaining its dynamic stability during the test), and the predominant influence stems from the muscle strength of the fingers, specifically when the assessment is conducted with the elbow flexed at 90° [11,28]. Understanding the specific muscles engaged in the HGS test and its protocol among athletes is essential to potentially explain the relationship between hand anthropometric variables and HGS from a biomechanical approach.

As mentioned before, finger muscles play a pivotal role during the execution of the HGS test [13,32]. For this reason, some authors [16] have suggested that athletes with longer fingers and greater hand surfaces exhibit higher HGS. However, these studies have primarily involved handball and basketball players, with the majority being male [13,16]. To our knowledge, there is a lack of evidence addressing this relationship in female volleyball players, making this study the first to elucidate it. Further research may provide more relevant evidence on this topic among female volleyball players across different competitive levels.

Finally, hand length, breadth, and hand shape index in both upper limbs demonstrated the strongest correlation with HGS (r = 0.32 to 0.66). These findings underscore the potential significance of these anthropometric parameters as a valuable predictor of HGS, demonstrating their practical applications of these variables as an essential, feasible, and cost-effective tool for helping coaches select athletes. Additionally, the HGS could be considered a measurement of preventing upper limb injuries, particularly in wrist and fingers, due to the specific actions during a match (i.e., blocking, serving, spiking)

This study has some strengths and limitations. Our sample of Latin American female volleyball players may not be considered representative of the entire population, even though it has proved sufficient to draw relevant conclusions. Furthermore, our sample consisted only of Latin American players. For that reason, additional studies could be developed in different populations to generate reference values that consider ethnicity and age characteristics. Some of the strengths of our study include the measurements of numerous anthropometric variables collected by an expert, the utilization of accurate and adequate tools, the adherence to a standardized HGS protocol based on consensus recommendations, and measurements of HGS conducted by an experienced researcher.

## 5. Conclusions

This study presents, for the first time, anthropometric and HGS parameters in Latin American female volleyball players. Particularly, this study provides evidence about the influence of anthropometric body and upper limbs (height, arm length, and hand dimension) on HGS and performance outcomes in sports requiring precise gripping and manipulative actions. Furthermore, this evidence highlights the importance of arm length and hand dimensions as basic and reliable measurements when HGS evaluation is not possible during the talent search process or characterization of female volleyball players. Future studies should explore the biomechanical parameters affecting HGS and consider their influences on athletic training and injury prevention strategies in female elite sports settings.

## Figures and Tables

**Table 1:** Descriptive statistics of HGS and some selected anthropometric characteristics in female volleyball players and controls.

Variables	Controls (*n* = 40)	Volleyball Players (*n* = 42)	Effect Size
Height (cm) ^+^	158.02 (6.02)	179.04 (12.0) ^†^	2.46
Body weight (kg)	57.21 (7.62)	71.12 (9.8) ^†^	1.57
Body mass index (kg/m^2^)	22.81 (2.91)	22.73 (2.3)	
Body fat (%)	25.23 (5.82)	22.22 (4.3) *	0.61
Body water (%) ^+^	52.01 (2.71)	53.11 (3.0) *	0.01
**Dominant upper limb**			
Upper arm length (cm)	30.01 (1.21)	34.51 (2.21) ^†^	2.40
Forearm length (cm)	23.44 (1.22)	25.84 (1.91) ^†^	1.49
Elbow diameter (cm)	6.21 (0.32)	6.21 (0.42)	
Wrist diameter (cm)	5.35 (0.31)	5.23 (0.33)	
Hand length (cm) ^+^	17.01 (1.02)	19.01 (1.52) ^†^	1.29
Hand breadth (cm) ^+^	7.32 (0.53)	7.53 (0.33) *	0.43
Hand length-breadth ratio ^+^	9.22 (0.52)	9.34 (0.52)	
Hand shape index ^+^	38.92 (3.54)	43.54 (2.63) ^†^	2.0
Handgrip strength (kg)	26.11 (3.92)	32.21 (6.31) ^†^	1.13
**Non-dominant upper limb**			
Upper arm length (cm)	29.81 (1.23)	33.61 (2.72) ^†^	1.76
Forearm length (cm) ^+^	23.02 (1.05)	25.63 (2.52) ^†^	1.31
Elbow diameter (cm)	6.01 (0.43)	6.03 (0.51)	
Wrist diameter (cm)	5.34 (0.24)	5.24 (0.32)	
Hand length (cm) ^+^	17.0 (0.53)	19.01 (1.84) ^†^	1.35
Hand breadth (cm) ^+^	7.31 (0.85)	7.31 (0.33)	
Hand length-breadth ratio ^+^	9.32 (0.34)	9.42 (0.54)	
Hand shape index ^+^	38.91 (3.62)	43.53 (2.64) ^†^	1.79
Handgrip strength (kg)	24.51 (4.32)	31.32 (5.91) ^†^	1.31

^+^ Data presented as median ± IQR; SD: standard deviation; * Significant at ≤0.05 level; ^†^ Significant at ≤0.01 level; Effect size is shown for statistically significance differences.

**Table 2:** Pearson’s correlation coefficients between anthropometric variables and dominant HGS in controls and Latin-American female volleyball players.

Dominant Handgrip Strength	Controls	Volleyball Players
Height (cm)	0.24	0.38 *
Dominant upper arm length (cm)	0.39 ^†^	0.38 *
Dominant forearm length (cm)	0.47 ^†^	0.26
Dominant elbow diameter (cm)	0.45 ^†^	0.32 *
Dominant wrist diameter (cm)	0.44 ^†^	0.22
Dominant hand length (cm)	0.38 *	0.43 *
Dominant hand breadth (cm)	0.34 *	0.63 ^†^
Hand length-breadth ratio	−0.15	−0.19
Hand shape index	−0.07	0.36 *

* Significant at ≤0.05 level; ^†^ Significant at ≤0.01 level.

**Table 3:** Pearson’s correlation coefficients between upper limb anthropometric variables and non-dominant HGS in Latin American female volleyball players and controls.

Non-Dominant Handgrip Strength	Controls	Volleyball Players
Height (cm)	0.07	0.29
Non-dominant upper arm length (cm)	0.35 *	0.34 *
Non-dominant forearm length (cm)	0.29	0.11
Non-dominant elbow diameter (cm)	0.38 ^†^	0.16
Non-dominant wrist diameter (cm)	0.29	0.34 *
Non-dominant hand length (cm)	0.26	0.32 *
Non-dominant hand breadth (cm)	0.36 ^†^	0.55 ^†^
Hand length-breadth ratio	−0.09	0.13
Hand shape index	0.11	0.35 *

* Significant at ≤0.05 level; ^†^ Significant at ≤0.01 level.

## Data Availability

The datasets used and/or analyzed during the current study are available from the corresponding author upon reasonable request.
